# New insights learned from the pulmonary to systemic blood flow ratio to predict the outcome in patients with hypoplastic left heart syndrome in the pre-Glenn stage: a single-center study

**DOI:** 10.3389/fcvm.2023.1207869

**Published:** 2023-08-03

**Authors:** Nathalie Mini, Peter A. Zartner, Martin B. E. Schneider

**Affiliations:** Department of Pediatric Cardiology, University Hospital Bonn, Bonn, Germany

**Keywords:** MBT shunt, hypo plastic left heart syndrome, Qp/QS, shunt stenosis, shunt stunting, pre stage II, Norwood Palliation

## Abstract

**Background:**

To the best of our knowledge, no study has been made until now to determine whether the ratio between pulmonary and systemic blood flow (*Q*p/*Q*s) in the pre-stage II (PS2) or pre-Glenn stage can predict the outcome in patients with hypoplastic left heart syndrome (HLHS) who underwent Norwood (NW) palliation.

**Patients and methods:**

From January 2016 to August 2022, 80 cardiac catheterizations in 69 patients with HLHS in NW palliation stage with modified Blalock–Taussig shunt (MBTS) were retrospectively recruited. The *Q*p/*Q*s was measured under stable conditions using the Fick formula. None of the patients were intubated. Patients were divided into two groups: Group 1 included patients who underwent planned cardiac catheterization (*n* = 56), and Group 2 had unplanned examination (*n* = 13), in which the indication for cardiac catheterization was desaturation in 11 patients and pulmonary over-circulation in two. The composite primary outcome was defined as accomplishing the planned operations (Glenn and Fontan) with freedom from death and reoperation, referring to palliative therapy or heart transplantation. The secondary outcome was freedom from transcatheter intervention in MBTS or pulmonary arteries.

**Results:**

The median follow-up was 48 months (range 6–72 months). The median value of *Q*p/*Q*s in Group 1 was 1.75 (range 1.5–2.2). In Group 2, the 11 patients with desaturation, the median value of *Q*p/*Q*s was 1.25 (range 0.9–1.45). The two patients with suspected pulmonary overcalculation showed *Q*p/*Q*s of 2.3 and 2.5, respectively; a reduction of the shunt size was required. Approximately 96.4% of patients in Group 1 achieved the primary outcome compared with only 30.7% in Group 2. The need for reintervention was 1.8% in Group 1 compared with 61.3% in Group 2. There is a significant relationship between *Q*p/*Q*s and the impaired outcome (death, palliative therapy, or heart transplantation) with a *p*-value of 0.001, a relative risk factor of 3.1, and a 95% confidence interval of 1.4–7.1. No significant relationship between the *Q*p/*Q*s and the size of MBTS (*p*-value of 0.073) was noted.

**Conclusion:**

The *Q*p/*Q*s in PS2 can predict outcomes in patients with HLHS in Norwood stage with MBTS. The *Q*p/*Q*s between 1.5 and 2.2 with a median of 1.75 seems to be optimal in the patients in PS2. *Q*p/*Q*s of <1.5 is associated with pulmonary stenosis, shunt stenosis, and pulmonary hypertension.

## 1. Introduction

Hypoplastic left heart syndrome (HLHS) is the most common lethal cardiac malformation in newborns. Norwood (NW) palliation stage I with either a modified Blalock–Taussig shunt (MBTS) or a Sano shunt is considered initial palliation in these patients. This procedure includes the connection of the divided main pulmonary artery to a reconstructed aortic arch, the creation of a shunt between the right subclavian artery and the pulmonary artery (MBTS), or a conduit between the right ventricle and the pulmonary artery (Sano shunt), ligation of the ductus arteriosus, and atrial septectomy. Several studies were undertaken to identify the optimal value of the pulmonary to systemic blood flow ratio (*Q*p/*Q*s), where the saturation and the hemodynamic situation were in the optimal range. Most of these studies were performed intraoperatively (1) and shortly after the operation during the hospital stay in the intensive care unit (ICU) (1–7).

Other studies (8, 9) have compared the hemodynamics between the Sano shunt and the MBTS in pre-stage II (PS2) palliation and showed that the *Q*p/*Q*s was lower in patients with the Sano shunt compared with those operated with MBTS (10). Migliavacca et al. (8, 9, 11) showed that in 2000 a *Q*p/*Q*s of 1 resulted in optimal O_2_ delivery in patients with a median age of 5 months.

Our current study attempts to determine whether the pulmonary to systemic blood flow ratio in PS2 can predict the outcome in the patients who underwent NW palliation with MBTS. In addition, we try to find the range of *Q*p/*Q*s values, in which the patients in this cohort were hemodynamically stable and highlight the pathologic values of *Q*p/*Q*s in some pathologic situations, such as pulmonary over-circulation, pulmonary hypertension (PHT), shunt stenosis, and pulmonary stenosis.

## 2. Patients and methods

Sixty-nine patients were retrospectively recruited in our center from 2016 to 2022. We performed 80 examinations on the 69 patients under sedation.

All cardiac catheterizations were done under sedation. Patients in whom the measurements were incomplete and those who suffered from sedation-related respiratory or hemodynamically compromise (*n* = 12), which can impact the outcome of the study, were excluded. Due to a low number of patients who were operated with a Sano shunt in our center from 2016 to 2022, they were also excluded from the current report.

The saturations were measured in the aorta (Ao-Sat), pulmonary vein (PV-Sat), inferior vena cava (IVC-Sat), and superior vena cava (SVC-Sat). The mixed venous saturation was measured as follows: SV-Sa = 3 × SVC + IVC/4. The pulmonary pressure was measured using an angiographic catheter, GLIDECATH® (Terumo, Radifocus GLIDECATH™, Non-taper Angle, 65 cm, 4 F), which was introduced through the shunt in the pulmonary arteries. The end-diastolic pressure of the systemic right ventricle was documented in all patients, as well as the hemoglobin (HB) and the hematocrit at the time of catheterization. The size of the MBTS, shunt stenosis, pulmonary stenosis, associated major aortopulmonary collateral arteries (MAPCAs), and the medication for heart failure therapy during cardiac catheterization (CC) were documented.

The examination was performed planned in 56 patients and unplanned in 13 due to desaturation (*n* = 11) or increased signs of pulmonary over-circulation (*n* = 2).

To analyze the optimal shunt flow, the patients in this study were divided into two groups: Group 1 includes patients who underwent a routine cardiac catheterization in preparing for the next step operation, and Group 2 includes patients who underwent an unplanned, more or less emergency, cardiac catheterization in PS2.

This study's composite primary outcome was freedom from all of the following: death, reoperation, referring to palliative therapy, or heart transplantation. The secondary outcome was freedom from transcatheter reintervention in MBTS or pulmonary arteries.

The *Q*p/*Q*s were measured using the Fick formula: Ao-Sat–SV-Sat/PV-Sat–PA-Sat, where SV-Sa = (3 × SVC + IVC)/4.

During follow-up, we documented the following events: death, need for a shunt stent, need for a shunt clip or shunt revision, time of Glenn and Fontan operation, and the need for heart transplantation or palliative therapy.

### 2.1. Statistical analysis

All statistical analyses were performed using SPSS version 22 (IBM). Continuous variables were reported as mean ± standard deviation (SD) and categorical variables as count (percentage). Non-paired Student’s *t*-test was used to compare the means of continuous variables between the two different categories. Chi-square test was used for comparing categorical variables. Odds ratios (ORs) ± 95% confidence intervals (95% CI) for the following parameters were calculated to assess any differences between Group 1 and Group 2: deaths in pre-Glenn stage (PGS) and the pre-Fontan stage, failing to arrive at the Glenn operation or the Fontan operation and being referred to palliative therapy or heart transplantation, need for shunt revision, or reoperation for pulmonary reconstruction or aortic arch reconstruction in PGS.

A *p*-value of 0.05 was set as the threshold for clinical significance. Kaplan–Meier survival curve analysis of the two different groups was performed.

### 2.2. Ethical statement

The study complies with the Declaration of Helsinki (as revised in 2013). Owing to a purely retrospective study design, using available institutional clinical records, with an absence of the impact on the management of the patients included and completely anonymous data presentation, informed consent of the subjects (or their parents) and ethical approval were not obtained.

## 3. Results

### 3.1. Group 1: patients who underwent planned cardiac catheterization in PS2

Sixty-five catheterizations were done electively in 56 patients as a routine examination in PS2 (Table 1). MBTS diameter was 4 mm in 15 patients and 3.5 mm in the rest of the patients. The median values of the age, body surface area (BSA), and weight were 4.1 months, 5 kg, and 0.31 m^2^, respectively. The median value of the *Q*p/*Q*s was 1.75, in which the median aortic saturation was 79.5%, and the mean hemoglobin was 12 g/dl. The median pulmonary artery pressure (mPAP) value was 12 mmHg, and the median value of end-diastolic pressure of the right ventricle (RV-EDP) was 9 mmHg. The median values of SVC-Sat, IVC-Sat, and PV-Sat were 48%, 52.3%, and 96%, respectively.

**Table 1:** Hemodynamic parameters in patients who underwent cardiac catheterization in the pre-Glenn stage.

Pat. Nr.	Planned CC	Unplanned CC due to desaturation^a^
	*n* = 56	*n* = 11
Median age (months)	4.1 (2–8)	5 (2–8)
Median weight (kg)	5 (3.8–9)	5.8 (3.8–11)
Median BSA (m^2^)	0.31 (0.24–0.35)	0.31 (0.24–0.35)
Median Ao-Sat (%)	79.5 (75–85)	75 (56–80)
Median SVC-Sat (%)	48 (35–63)	52 (33–63)
Median PV-Sat (%)	96 (91–98)	95 (91–98)
Median HB (g/dl)	12 (11–17)	13 (10.7–15)
Median *Q*p/*Q*s	1.75 (1.5–2.2)	1.25 (0.9–1.45)
Median mPAP (mmHg)	12 (9–14)	15.5 (11–23)
Median EDP (mmHg)	9 (6–12)	12.6 (7–20)
Median PVRI (Wum²)	0.64 (0.48–1.2)	1.35 (0.7–2.1)

PVRI, pulmonary vascular resistance index.

Values in parentheses indicate ranges.

^a^ Thirteen patients underwent unplanned CC (two of them underwent CC due to heart failure with *Q*p/*Q*s of 2.3 and 2.5, respectively).

In six patients, the MAPCAs needed to be occluded with coils, and no change in the *Q*p/*Q*s before and after the occlusion was documented. All patients became the standard medication of our center after NW palliation at the time of catheterization, which includes beta-blocker and cardiac glycoside (digoxin). The need for increased doses of beta-blocker and digoxin was noticed in two patients, in whom *Q*p/*Q*s was 2.1 and 2.2, respectively.

One patient needed shunt stenting due to apparent shunt stenosis without notable desaturation.

The median follow-up in this group was a median of 48 (range 6–72) months. In midterm and long-term follow-ups, two deaths were documented. The first patient had Kabuki syndrome. Due to his long-term stay in the ICU postoperatively and his syndrome, he was not discharged from the hospital. The Glenn operation was not amenable during the hospital stay due to a chronic Cytomegalovirus (CMV) infection. He died 5 months after the NW procedure because of respiratory failure. The second patient had a 3.5 MBTS implanted and was successfully brought to Glenn operation. He suddenly died at home after an infection at 3 years old.

### 3.2. Group 2: patients who underwent unplanned cardiac catheterization in PS2

Twenty unplanned examinations were performed on 13 patients. The indication for cardiac catheterization was desaturation in 11 patients and increased signs of pulmonary over-circulation in two. The median age, weight, and BSA values were 5 months, 5.8 kg, and 0.31 m², respectively. The size of the MBTS was 3.5 mm in seven patients and 4 mm in six.

#### 3.2.1. Unplanned catheterization due to desaturation

Sixteen examinations were unplanned in 11 patients due to desaturation (Table 1). The diameter of the MBTS was 3.5 mm in seven patients and 4 mm in four.

The median value of the *Q*p/*Q*s was 1.25, in which the mean aortic saturation was 75% by mean hemoglobin of 13 g/dl. The mPAP value was 15.5 mmHg, and the median value of RV-EDP was 12.6 mmHg. The median values of SVC-Sat, IVC-Sat, and PV-Sat were 52%, 52%, and 95%, respectively. Six patients had shunt stenosis, five needed shunt stenting, and one was operated on with the Glenn procedure 2 days after the catheterization. One patient had severe stenosis of the truncus brachiocephalic entrance, which needed to be stented; later, the patient received a central shunt due to restenosis.

We have documented associated pulmonary stenosis in five patients and pulmonary hypertension (18–31 mmHg) in four.

In follow-up, four deaths were documented in PS2 due to shunt occlusion in one patient (6 months old) with multiple thrombotic events and a stent in the shunt. Sudden death was recorded in the second patient (8 months old) with a stent in the shunt in whom shunt occlusion was highly expected to cause the death. The third death was documented in a patient (6 months old) with PHT in PS2, and the fourth death was in a patient with severe stenosis in the truncus brachiocephalicus (TBC), which was treated with a stent. Because of the restenosis, the shunt was revised to a central shunt. The patient was then operated on with a right Glenn (to the right pulmonary artery) and a left shunt (to the left pulmonary artery); the patient died in a palliative care center at 12 months.

#### 3.2.2. Unplanned catheterization in PGS due to cardiac insufficiency

Two patients were referred to cardiac catheterization due to increased signs of pulmonary over-circulation: sweating, tachypnea, failure to gain weight, elevated saturation (>85%), and excessive diuretic need. The first patient had a 4 mm MBTS implanted, which was mildly clipped. The cardiac catheterization showed *Q*p/*Q*s of 2.3 and severe stenosis in the aortic arch. Echocardiographic examination showed significant tricuspid regurgitation. The patient was referred for surgery and had a reconstruction of the aortic arch and the tricuspid valve. Three months after the tricuspid valve reconstruction, the patient died at 7 months due to cardiac decompensation and had not received the Glenn operation. The MBTS in the second patient was 4 mm, the *Q*p/*Q*s was 2.5, and a shunt clip was required. The patient was operated on during follow-up, and a Glenn anastomosis was established. At this stage, the patient developed severe heart failure due to massive collateral artery flow (3–4 mm in diameter) and significantly reduced RV ejection fraction (EF of <25%). The *Q*p/*Q*s in this Glenn stage was 0.8. After most of the MAPCAs were closed, the echocardiography showed only moderately reduced RV-EF, and the patient is awaiting a Fontan operation.

### 3.3. Morbidity and mortality

There is a significant relationship between death, failure to reach the Glenn operation, being referred to a palliative therapy or HTx, and *Q*p/*Q*s ratios with a *p*-value of 0.001 with a relative risk factor (RR) of 3.1 and a 95% CI of 1.4–7.1 (odds ratios: 65; 95% CI 10.4–409).

## 4. Discussion

The challenge after the Norwood palliation with MBTS is to reach a balance between the pulmonary circulation and the systemic circulation to avoid pulmonary over-circulation, unbalanced high systemic oxygen delivery, and the negative effect of the aortic diastolic runoff on coronary perfusion, which is one of the leading causes of morbidity and mortality early after the palliation (5, 6). Many studies were published for a better understanding of the hemodynamics of Norwood palliation in patients with HLHS intraoperatively and shortly after the operation. Some results suggested that the *Q*p/*Q*s should be over 1.5 for an improved course early after the palliation stage (6). Another found that the circulation in these patients was balanced when the *Q*p/*Q*s is equal to 1.4:1 (3).

Other authors compared the hemodynamics between the Norwood palliation stage I operation with MBTS, the Sano shunt in the early stage after Norwood palliation, and the PGS. The results showed that the *Q*p/*Q*s was higher in patients with MBTS than in those who underwent Sano shunt. The end-diastolic RV pressure was lower in the Sano shunt compared with the MBTS (8, 9).

In the current report, we have focused on the patients who underwent Norwood with MBTS in PS2, trying to find the optimal *Q*p/*Q*s ratios at which hemodynamic stability was observed and analyzing if the hemodynamics of the patients who underwent catheterization in PS2 can predict the outcome in the pre-Fontan stage (PS3).

The current study showed that the patients in Group 1 who had *Q*p/*Q*s between 1.5 and 2.2 (median 1.75) were clinically stable with adequate saturation and had no or minimal cardiac insufficiency compared with those in Group 2 who had *Q*p/*Q*s under 1.5 or more than 2.2. The mean pressure of the pulmonary artery and the RV-EDP were higher in Group 2 compared with patients in Group 1. The need for shunt or pulmonary stenting, revision of a shunt, shunt clips, or pulmonary artery enlargement or aortic arch reconstruction in PGS was 69% compared with 1.6% in Group 1. In a follow-up with a median of 48 months, 54% of patients in Group 2 died in PGS or were referred to palliative palliation compared with 3.3% in Group 1.

**Figure 1:**
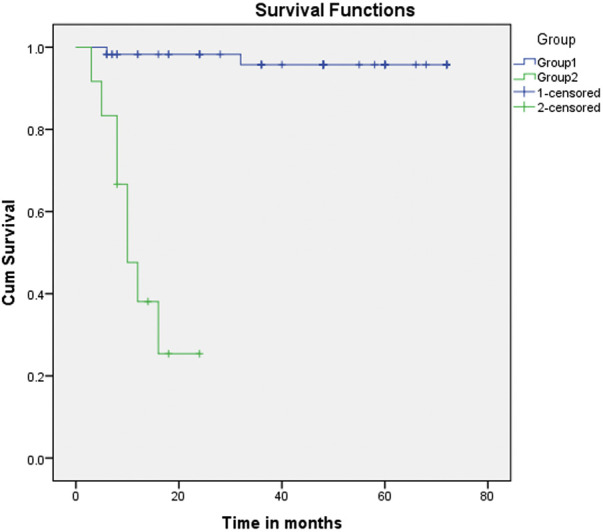
Kaplan–Meier curve (primary outcome: Group 1 vs. Group 2). Kaplan–Meier curve (primary outcome: Group 1 vs. Group 2). Group 2 showed a meager survival compared with Group 1. Patients with *Q*p/*Q*s between 1.5 and 2.2 had much better survival rates, with the majority reaching the Fontan operation. Time since the patients received the NW procedure in months.

**Figure 2:**
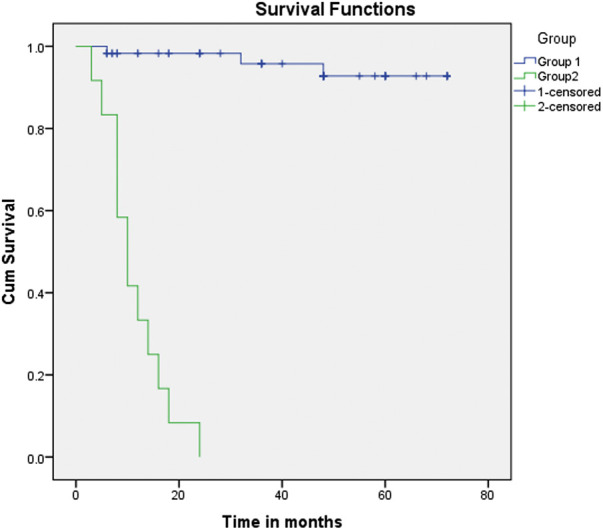
Kaplan–Meier curve (primary and secondary outcome: Group 1 vs. Group 2). Kaplan–Meier curve (primary and secondary outcome: Group 1 vs. Group 2). Group 2 showed very low survival rates and an increased need for interventions compared with Group 1 (*Q*p/*Q*s: between 1.5 and 2.2).

This study showed a significant relationship between morbidity and mortality and *Q*p/*Q*s. The survival analysis (Figures 1 and 2) demonstrates an explicit disassociation between the two groups. No significant relationship between *Q*p/*Q*s and the size of shunts was reported.

A need for shunt stenting related to shunt stenosis was observed in patients with *Q*p/*Q*s of <1.5, in whom the desaturation indicated catheterization in PS2. This observation was also documented in patients with severe pulmonary stenosis. It is worse to notice that the patients with pulmonary hypertension in PS2 showed a low *Q*p/*Q*s and had failed to reach the stage of Glenn. Severe heart failure with pulmonary over-circulation and the need for shunt clips were observed in two patients with *Q*p/*Q*s of >2.2.

Although some centers seek to replace the routine cardiac catheterization before Glenn with MRI to evaluate the development of pulmonary arteries and lymphatic system and calculate the *Q*p/*Q*s, MRI cannot calculate pulmonary vascular resistance, which is essential for evaluating the conditions in PS2 and PS3. However, the calculation of *Q*p/*Q*s in cardiac catheterization labor could be limited in patients with sedation-related respiratory or hemodynamic instability or those needing respiratory support or medication which impact the hemodynamic situation or the pulmonary vascular resistance.

## 5. Conclusion

*Q*p/*Q*s ratios in the PS2 in patients with HLHS who underwent Norwood palliation stage I with MBTS can predict the outcome of these patients. The optimal *Q*p/*Q*s ratios in PS2 in our cohort range between 1.5 and 2.2 with a median of 1.75 as morbidity and mortality were observed to be significantly higher if *Q*p/*Q*s is outside these limits.

## Data Availability

The original contributions presented in the study are included in the article, further inquiries can be directed to the corresponding author.
